# Early postpartum dysglycemia after gestational diabetes: β-cell dysfunction and risk stratification in a multiethnic cohort

**DOI:** 10.3389/fendo.2026.1913417

**Published:** 2026-08-12

**Authors:** Pei Chia Eng, Ryan Jinn Tan, Rui Cheng Luo, Amal Al-Amri, Lyeann Yi Ling Tan, Chioma Izzi-Engbeaya, Chin Meng Khoo

**Affiliations:** 1 Section of Investigative Medicine and Endocrinology, Department of Metabolism, Digestion and Reproduction, Imperial College London, London, United Kingdom; 2 Division of Endocrinology, Department of Medicine, National University Hospital, Singapore, Singapore; 3 Lee Kong Chian School of Medicine, Nanyang Technological University, Singapore, Singapore; 4 Department of Internal Medicine, Nizwa Hospital, Oman, Oman

**Keywords:** Asian phenotype, body mass index, gestational diabetes, oral glucose tolerance test, postpartum *dysglycemia*

## Abstract

**Introduction:**

Postpartum oral glucose tolerance test (OGTT) screening is recommended after gestational diabetes (GDM), but attendance is often incomplete and testing may not capture metabolic heterogeneity. Western-derived risk models may be less applicable to Asians, in whom *dysglycemia* may occur at lower body mass index (BMI) and reflect greater β-cell vulnerability. We examined the prevalence, clinical phenotype and predictors of early postpartum *dysglycemia* in women with GDM.

**Methods:**

We retrospectively studied 575 women with GDM managed at a tertiary center in Singapore between 01/10/2023 to 30/04/2025. Early postpartum *dysglycemia* was defined by World Health Organization criteria using 6–8 weeks postpartum OGTT. Multivariate logistic regression identified predictors of postpartum dysglycemia. Missing data were handled by multiple imputation; candidate predictors were screened by univariate logistic regression and assessed for multicollinearity using variance inflation factors. Model performance was evaluated by area under the receiver operating characteristics curve (AUC), with internal validation using 1000 bootstrap resamples. Dominance analysis quantified relative predictor contributions.

**Results:**

Postpartum OGTT completion rate was 71.1% (409/575). Among women tested, 34.2% (140/409) had early postpartum dysglycemia. Dysglycemia occurred in a relatively lean phenotype and was associated with lower homeostatic model assessment of β-cell function (HOMA-β). Independent predictors were family history of diabetes, prior GDM, elevated pregnancy 2-hours OGTT glucose, Chinese ethnicity, and higher HbA1c while *in-vitro* fertilization and higher pre-pregnancy BMI were inversely associated with dysglycemia. Discrimination was good with AUC 0.77 (95% CI 0.72 – 0.81). Dominance analysis ranked pregnancy 2-hour OGTT glucose (18.8%) and family history (17.1%) as top contributors. Threshold analyses suggested pregnancy 2-hours OGTT ≥8.5 mmol/L was specific but insensitive, whereas antepartum HbA1c ≥5.5% was sensitive but less specific.

**Conclusions:**

Early postpartum dysglycemia after GDM was common and appeared consistent with a relatively lean, β-cell–predominant phenotype. Pragmatic risk stratification may complement tiered postpartum surveillance and prevention.

## 1 Introduction

Gestational diabetes mellitus (GDM), defined as glucose intolerance first recognized during the second or third trimester of pregnancy (1), has risen in parallel with the global obesity and diabetes epidemics (2). Using the 2013 World Health Organization (WHO) diagnostic criteria (3), the global prevalence of GDM is ~14% with substantial geographical variation. The estimated prevalence is approximately ~20.8% in Southeast Asia compared with ~7.8% in Europe (4). Beyond the well-recognized association of GDM with maternal and neonatal complications such as preeclampsia, preterm birth, caesarean delivery and fetal macrosomia (5), GDM is also a predictor of metabolic risk after pregnancy (6). Women with prior GDM have a ten-fold increased future risk of developing type 2 diabetes (T2DM) (7), with the risk accruing most rapidly in the first five years postpartum (8) at an estimated rate of 9.6% per year (9). Therefore, the early postpartum period represents a clinically important window for risk stratification and initiation of targeted prevention to attenuate the progression to T2DM and its associated cardiometabolic complications.

Current guidelines recommend reassessment of glycemic status with a 75g 2 hours-oral glucose tolerance test (OGTT) within the first 3 to 6 months postpartum in women with GDM after delivery (10–12). This approach classifies women into impaired fasting glucose (IFG), impaired glucose tolerance (IGT), diabetes mellitus, or normal glucose tolerance (NGT). IFG and IGT, often grouped as “prediabetes”, represent intermediate stages between NGT and overt diabetes and are characterized by insulin resistance accompanied by impaired β-cell function (13). While this single time-point reassessment of glycemic status may appear clinically useful, it often fails to identify a sizeable proportion of high-risk women. This is because postpartum glycemia exists on a continuum and trajectories can evolve rapidly. Retnakaran et al. observed that 17% of women with prior GDM who had NGT at 3 months postpartum progressed to dysglycemia within one year after index pregnancy (14). Furthermore, women with mild glucose intolerance below the diagnostic threshold for GDM were also observed to have persistent β-cell dysfunction at 3 to 12 months after delivery (15). Importantly, this metabolic heterogeneity appears to vary in different populations. In our previous multiethnic Asian cohort, women who developed postpartum dysglycemia exhibited higher antepartum glycemic indices despite lower pre-pregnancy body mass index (BMI) compared to women with NGT (16). Compared with Western populations, Asian individuals may have greater visceral adiposity at a given BMI and develop dysglycemia at lower BMIs (17, 18). Reduced β-cell reserve has also been shown to contribute to dysglycemia in Chinese and Indian populations. Among Chinese women with previous GDM, β-cell dysfunction was more strongly associated with postpartum diabetes in women without obesity, whereas insulin resistance was more prominent in women with obesity (19). These observations suggest that reliance on a single postpartum OGTT, or on BMI alone, may not be sufficient to risk-stratify patients to guide personalized intervention and diabetes prevention. However, validated prediction models for postpartum dysglycemia in multiethnic Asian populations remain limited.

In parallel, uptake of postpartum OGTT has remained suboptimal globally with completion rates ranging between 31% to 49% (20–22), despite a near universal antenatal screening rate (98%) (23). In our institution, postpartum follow-up is embedded into standard obstetric care. However, even within this structured clinical pathway, non-attendance remains an issue because women at highest metabolic risk are least likely to adhere to postpartum OGTT testing (16). In this context, risk prediction using routinely available clinical and biochemical data from the antepartum and early postpartum stages could provide a scalable complement to universal OGTT, supporting a tiered follow-up (e.g. prioritized reminders, earlier appointments, streamlined testing alternatives where OGTT is not feasible) and improve efficiency in postpartum services.

Accordingly, we aim to first, quantify the prevalence and characterize metabolic phenotype of early postpartum dysglycemia following GDM in a multiethnic Asian cohort, and second, to develop a reliable prediction model using clinically available variables. We additionally quantify the relative contribution of predictors to support practical triage within postpartum care pathways.

## 2 Methods

We conducted a retrospective cohort study based on data collected from a clinical service for all women diagnosed with GDM from 01/10/2023 to 30/04/2025 at a tertiary center in Singapore. GDM was diagnosed using a 75g OGTT performed after an overnight fast (8 to 10 hours) using the 2013 WHO criteria (fasting plasma glucose [FPG] ≥ 5.1 mmol/L [92 mg/dL] and/or 1-hour plasma glucose ≥ 10.0 mmol/L [180 mg/dL] and/or 2-hour plasma glucose ≥ 8.5 mmol/L [153 mg/dL]) at 24 to 28 weeks gestation) (3). At 6 to 8 weeks after delivery, women with GDM in our institution were routinely offered a repeat 75g OGTT as recommended by local guidelines (24). These 6–8 weeks timing for OGTT testing were chosen to align with when patients are seen in the obstetrics & gynecology clinic for their obstetric postnatal follow-up. At our institution, women with GDM are offered an additional follow-up appointment at the outpatient endocrinology clinic at 8 to 12 weeks postpartum (postpartum endocrine clinic). During the postpartum endocrine visit, OGTT results are reviewed, and women receive personalized counselling on healthy diet, physical activity, breastfeeding sleep and long-term diabetes prevention. The postpartum OGTT classifies women into categories such as IGT, IFG, or T2DM. Criteria for diagnosis are as follows: (1) IFG defined as FPG ≥6.1 to <7 mmol/L (110–126 mg/dL) (2) IGT defined as FPG <7.0 mmol/L (126 mg/dL) and 2-hour glucose ≥7.8 to <11.1 mmol/L (140–199 mg/dL), or HbA1c between 42 and 48 mmol/mol (5.7–6.4%) (3) T2DM defined as FPG ≥ 7.0 mmol/L (126 mg/dL), 2-hours OGTT glucose ≥11.1 mmol/L (200 mg/dL), or HbA1c ≥48 mmol/mol (≥ 6.5%). Women with twin pregnancies, pre-existing type 1 or type 2 diabetes mellitus or women who use medications that affect glucose tolerance (e.g. systemic corticosteroids) or those who had abnormal *hemoglobin* variants on *hemoglobin* electrophoresis that could interfere with glycated *hemoglobin* (HbA1c) measurement were excluded from this study.

Antepartum parameters collected include self-reported ethnic background, maternal age at GDM pregnancy, gravidity, parity, family history of diabetes in first-degree relatives, pre-existing diagnosis of polycystic ovary syndrome (PCOS) or previous GDM, pre-pregnancy BMI/weight, treatment received during pregnancy (diet, metformin and/or insulin), glucose values from antenatal (24 to 28 weeks) diagnostic OGTT, HbA1c at 24 to 28 weeks and mode of delivery. Postpartum clinical variables collected include postpartum OGTT glucose values, body weight and BMI, C-peptide, HbA1c, lipids [total cholesterol, triglycerides, high-density lipoprotein (HDL), and low-density lipoprotein (LDL)], liver enzymes, and gestational weight gain (GWG) were classified based on recommendations by the Institute of Medicine (IOM) (25). C-peptide concentrations were determined by chemiluminescence immunoassays (ADVIA Centaur XP; Siemens, Germany). The assay demonstrated intra-assay coefficient of variance (CV) of 3.7% to 4.7% and inter assay CV of 5.1 to 9.5%. HbA1c was measured by cation-exchange high performance liquid chromatography (26). Concentrations of total plasma triglyceride, total cholesterol, cholesterol in low- and high-density lipoproteins (LDL and HDL, respectively), plasma glucose during the OGTT, and alanine aminotransferase were determined by the AU5822 general chemistry analyzer (Beckman Coulter, CA, USA) at the National University Hospital Referral Laboratory (accredited by the College of American Pathologists). Insulin resistance (IR) and β-cell function (B) were assessed using the homeostatic model assessment indices HOMA-IR and HOMA-β, calculated from postpartum fasting C-peptide (FCP) and FPG concentrations using the following equations (27): HOMA-IR = 1.5 + [(FPG x FCP)/2800], HOMA-β = (0.27 x FCP)/(FPG-3.5). We classified prediabetes as women who had IFG, IGT, or combined IFG/IGT on a postpartum OGTT test, and our outcome measure is postpartum dysglycemia, which refers to women with prediabetes and or T2DM.

### 2.1 Statistical analyses

All statistical analyses were performed using R version 4.4.1. Continuous variables are presented as median (25^th^ percentile, 75^th^ percentile). Categorical variables are presented as n/N (%), where N denotes the number of participants with available data after excluding missing values. Between-group comparisons for descriptive analyses were performed using the Mann–Whitney U test for continuous variables and the χ^2^ or Fisher’s exact test for categorical variables, as appropriate.

Missing variables were evaluated prior to regression analysis. Multiple imputation by chained equations (MICE) (28) was applied for variables with <20% missing data. HbA1c at 24 to 28 weeks, with 33.7% missingness was also imputed using MICE given its clinical relevance. The imputation model included postpartum glycemic status and the candidate predictors considered in the regression analysis, including maternal age, ethnicity, family history of diabetes, previous GDM, IVF conception, pre-pregnancy BMI, antenatal fasting, 1-hour and 2-hour OGTT glucose concentrations, antepartum HbA1c, and GDM treatment. Imputation was performed under a missing-at-random assumption, whereby missingness was assumed to be explainable by the observed variables included in the imputation model. Univariate logistic regression was performed for all imputed candidate predictors and multicollinearity was assessed using variance inflation factors (VIF) (29). Predictors with p < 0.05 and VIF < 5 were retained and entered into a multivariate logistic regression model, which was refined using Akaike Information Criterion (AIC) (30).

Odds ratios (ORs) with 95% confidence intervals (CIs) were estimated from the final multivariate logistic regression model. Model discrimination was evaluated using area under the receiver operating characteristics curve (AUC) with internal validation performed using 1,000 bootstrap resamples. Dominance analysis was used to assess the mean contribution of each predictor to McFadden’s pseudo-R-squared (31) in the final multivariate logistic regression model. Bootstrapping with 1000 resamples was conducted for internal validation. Mean contributions and 95% CIs were then normalized to percentages for data interpretability. For significant continuous predictors, optimal cut-off points were determined via Youden’s index, which identifies the threshold that maximizes sensitivity and specificity (32). Statistical significance was set at p < 0.05.

## 3 Results

### 3.1 Antepartum characteristics of women with GDM stratified by postpartum glycemic status

The antepartum characteristics of women with GDM stratified by postpartum glycemic status are summarized in **Table 1**. A total of 575 women diagnosed with GDM received care at our center during the study period, however, only 409 women completed the postpartum OGTT. The remaining 166 women did not complete postpartum testing within the clinical follow-up period and were therefore not included in the analysis. As this was a retrospective study of routine clinical care, no study-directed follow-up or additional outcome ascertainment was performed for women who did not return for postpartum testing. Among the 409 women with prior GDM, 140/409 (34.2%) had postpartum dysglycemia and 269/409 (65.8%) had normal glucose tolerance (NGT). The distribution of ethnic backgrounds differed between groups, with a higher proportion of Chinese women with postpartum dysglycemia (62.1%) compared to the other ethnic groups (p=0.00). Age at presentation, gravidity, and parity were comparable in the postpartum dysglycemia and NGT groups. A family history of diabetes was more common in women with postpartum dysglycemia (60% vs 41.8%, p=0.00), whereas all other antepartum parameters were comparable. Conception by *in-vitro* fertilization (IVF) was less frequent in the postpartum dysglycemia group than in the NGT group (2.9% vs 9%, p=0.04). Women who conceived by IVF were older with lower pre-pregnancy weight/BMI (**Supplementary Table 1**). Women who had postpartum dysglycemia also had higher antepartum HbA1c (5.3% vs 5.1%, p=0.00) and 2-hour OGTT glucose level (9.3 mmol/L vs 8.6 mmol/L, p=0.00). There was a trend towards higher 1-hour OGTT glucose level (10.3 mmol/L vs 10.1 mmol/L, p=0.05) and no difference in fasting antepartum OGTT glucose level (4.6 mmol/L vs 4.6 mmol/L, p=0.15). Despite higher antepartum glycemic indices, women with postpartum dysglycemia had lower pre-pregnancy and mid-pregnancy (24 to 28 weeks) weight and BMI compared to women with NGT (**Table 1**), while weight gain and BMI change over pregnancy were comparable in both groups. Compared with non-completers, women who completed the postpartum OGTT were more often Chinese, had lower pre-pregnancy BMI, and more frequently reported a family history of diabetes, while non-completers had slightly higher antepartum HbA1c (**Supplementary Table 2**). Other characteristics were similar between groups.

**Table 1:** Baseline maternal demographic and antepartum clinical characteristics of women with gestational diabetes mellitus, stratified by postpartum glycemic status.

Antepartum variables	Postpartum glycemic status		p-value
	Dysglycemia (n = 140)	NGT (n = 269)	
Age at GDM pregnancy (years)	34 (31, 37)	33 (30, 36)	0.10
Race			
Chinese	87/140 (62.1)	117/268 (43.7)	0.00
Malay	36/140 (25.7)	95/268 (35.4)	
Indian	10/140 (7.1)	29/268 (10.8)	
Others	7/140 (5)	27/268(10.1)	
Family history of diabetes	78/130 (60)	99/237 (41.8)	0.00
PMH of GDM	41/140 (29.3)	54/262 (20.6)	0.07
PMH of gestational hypertension	1/105 (1)	7/205 (3.4)	0.36
PMH of PCOS	6/137 (4.4)	10/243 (4.1)	1.00
IVF status	4/139 (2.9)	24/267 (9)	0.04
Gravidity	2 (1, 3)	2 (1, 3)	0.68
Parity	1 (1, 2)	1 (0, 1)	0.33
Pre-pregnancy weight (kg)	59.9 (53, 70.8)	64 (54, 75.6)	0.03
Pre-pregnancy BMI (kg/m^2^)	24 (21, 28.7)	25.4 (22.2, 30.1)	0.04
Weight at 24–28 weeks (kg)	65.1 (57.5, 75.1)	69.5 (60.9, 80.1)	0.01
BMI at 24–28 weeks (kg/m^2^)	26 (23.3, 31.1)	27.8 (24.6, 32)	0.02
Treatment for GDM			
Diet	87/134 (64.9%)	199/267 (74.5%)	0.12
Metformin	6/134 (4.5%)	61/267 (22.8%)	
Insulin	39/134 (29.1%)	5/267 (1.9%)	
Insulin and Metformin	2/134 (1.5%)	2/267 (0.7%)	
Change in weight over pregnancy (kg)	9 (5.3, 11.4)	9 (4.8, 12.5)	0.94
Change in BMI over pregnancy (kg/m^2^)	3.5 (2.3, 4.8)	3.5 (1.8, 5)	0.89
HbA1c at 24 to 28 weeks (%)	5.3 (5, 5.6)	5.1 (4.9, 5.4)	0.00
Baseline glucose during OGTT at 24–28 weeks (mmol/L)	4.6 (4.2, 5.2)	4.6 (4.2, 5)	0.15
1-hour glucose during OGTT at 24–28 weeks (mmol/L)	10.3 (9.38, 11.1)	10.1 (9.2, 10.8)	0.05
2-hour glucose during OGTT at 24–28 weeks (mmol/L)	9.3 (8.5, 10)	8.6 (7.7, 9.4)	0.00

BMI, body mass index; GDM, gestational diabetes mellitus; HbA1c, glycated *hemoglobin*; IVF, *in vitro* fertilization; NGT, normal glucose tolerance; OGTT, oral glucose tolerance test; PCOS, polycystic ovary syndrome; PMH, past medical history.

Dysglycemia refers to abnormal glucose tolerance on postpartum OGTT, including impaired fasting glucose, impaired glucose tolerance or diabetes mellitus. Continuous variables are presented as median (25^th^ percentile, 75^th^ percentile). Categorical variables are presented as n/N (%), where N denotes the number of participants with available data after excluding missing values. Group comparisons were made using the Mann–Whitney U test for continuous variables and the χ² or Fisher’s exact test for categorical variables, as appropriate. Statistical significance was set at p < 0.05.

### 3.2 Postpartum characteristics of women with GDM stratified by postpartum glycemic status

The median time from delivery to postpartum OGTT was 48 (44, 67) days. Of the 409/575 (71.1%) who attended the postpartum OGTT, 140 (34.2%) had dysglycemia and 269 (65.8%) NGT. Among the women classified as being dysglycemic, 13 (9.3%) had IFG, 93 (66.4%) had IGT, 6 (4.3%) women had both IFG and IGT, and 28 (20%) had T2DM on postpartum OGTT. Women with postpartum dysglycemia had higher postpartum HbA1c (5.5% vs 5.4% p<0.005) (**Table 2**), lower weight at delivery (69 kg vs 72.6 kg, p<0.005) and at first clinic visit (61.4 kg vs 66.1 kg, p=0.03). BMI at delivery was also lower at the first postpartum clinic visit in women with dysglycemia (24.4 kg/m^2^ vs 26.6 kg/m^2^, p=0.01), with differential distribution of weight categories in women of different ethnic backgrounds (**Supplementary Table 3**). Waist-to-hip ratio, low-density lipoprotein (LDL), triglycerides, liver enzymes, C-peptide, or HOMA-IR levels showed no significant differences between groups. However, HOMA-β was significantly lower in women with postpartum dysglycemia compared to NGT (0.3 vs 0.5, p<0.005) (**Table 2**). All women with GDM delivered at a median of 38 (37, 39) weeks of gestation, and baby birthweight was comparable in both groups.

**Table 2:** Postpartum characteristics of women with GDM stratified by postpartum glycemic status. .

Postpartum variables	Postpartum glycemic status		p-value
	Dysglycemia (n = 140)	NGT (n = 269)	
Gestational age at delivery (weeks)	38 (37, 39)	38 (37, 39)	0.65
Baby birthweight (g)	2950 (2545, 3265)	3030 (2700, 3310)	0.16
Time to postpartum OGTT from delivery (days)	47 (43, 63)	48 (44, 68)	0.07
Postpartum fasting glucose (mmol/L)	4.9 (4.5, 5.5)	4.7 (4.4, 5)	0.00
Postpartum HbA1c (%)	5.5 (5.3, 5.8)	5.4 (5.1, 5.6)	0.00
Weight at delivery (kg)	69 (61.8, 82.2)	72.6 (64, 84.8)	0.05
BMI at delivery (kg/m^2^)	27.8 (25.2, 32.6)	29.4 (25.7, 33.4)	0.05
Fasting glucose at first clinic visit (mmol/L)	5 (4.5, 5.5)	4.8 (4.4, 5.1)	0.02
Time to first postpartum clinic from delivery (days)	85 (65, 104)	91 (72, 119)	0.09
Weight at first clinic visit (kg)	61.4 (52.9, 74.2)	66.1 (58, 75.9)	0.03
BMI at first clinic visit (kg/m^2^)	24.4 (20.8, 29.3)	26.6 (23.2, 30)	0.01
Waist-hip ratio at first clinic visit	0.8 (0.8, 0.9)	0.8 (0.8, 0.9)	0.95
Hb at first clinic visit (g/dL)	12.6 (11.6, 13.4)	12.5 (11.1, 13.2)	0.20
Platelets at first clinic visit (10^9^/L)	274 (238, 328)	274 (218.6, 318)	0.55
Total cholesterol at first clinic visit (mmol/L)	5 (4.5, 6)	5.05 (4.7, 5.7)	0.79
Triglycerides at first clinic visit (mmol/L)	0.9 (0.6, 1.4)	1.1 (0.7, 1.6)	0.06
LDL at first clinic visit (mmol/L)	3.1 (2.6, 3.7)	3.1 (2.7, 3.7)	0.64
HDL at first clinic visit (mmol/L)	1.5 (1.3, 1.9)	1.4 (1.1, 1.6)	0.01
ALT at first clinic visit (units/L)	23 (17, 31)	22 (14.8, 35)	0.67
AST at first clinic visit (units/L)	23 (20, 28)	24 (20, 28)	0.80
ALP at first clinic visit (units/L)	79.5 (66.8, 98.3)	85 (70, 102)	0.35
Albumin at first clinic visit(g/L)	45 (43, 46)	44 (42, 46)	0.00
C-peptide at first clinic visit (pmol/L)	515 (356, 870)	696 (426, 948)	0.14
HOMA-IR	1.6 (1.5, 1.6)	1.6 (1.5, 1.6)	0.49
HOMA-β	0.3 (0.2, 0.5)	0.5 (0.3, 0.7)	0.00

ALP, alkaline phosphatase; ALT, alanine aminotransferase; AST, aspartate aminotransferase; BMI, body mass index; Hb, *hemoglobin*; HDL, high density lipoprotein; HOMA-β, homeostatic model assessment of β-cell function; HOMA-IR, homeostatic model assessment of insulin resistance; LDL, low density lipoprotein; NGT, normal glucose tolerance.

Dysglycemia refers to abnormal glucose tolerance on postpartum oral glucose tolerance test (OGTT) and included impaired fasting glucose, impaired glucose tolerance or diabetes mellitus. Data are presented as median (25^th^ percentile, 75^th^ percentile) unless otherwise stated. P-values represent between-group comparisons using the Mann–Whitney U test. Statistical significance was set at p < 0.05.

### 3.3 Multivariate regression model for postpartum dysglycemia

**Figure 1:**
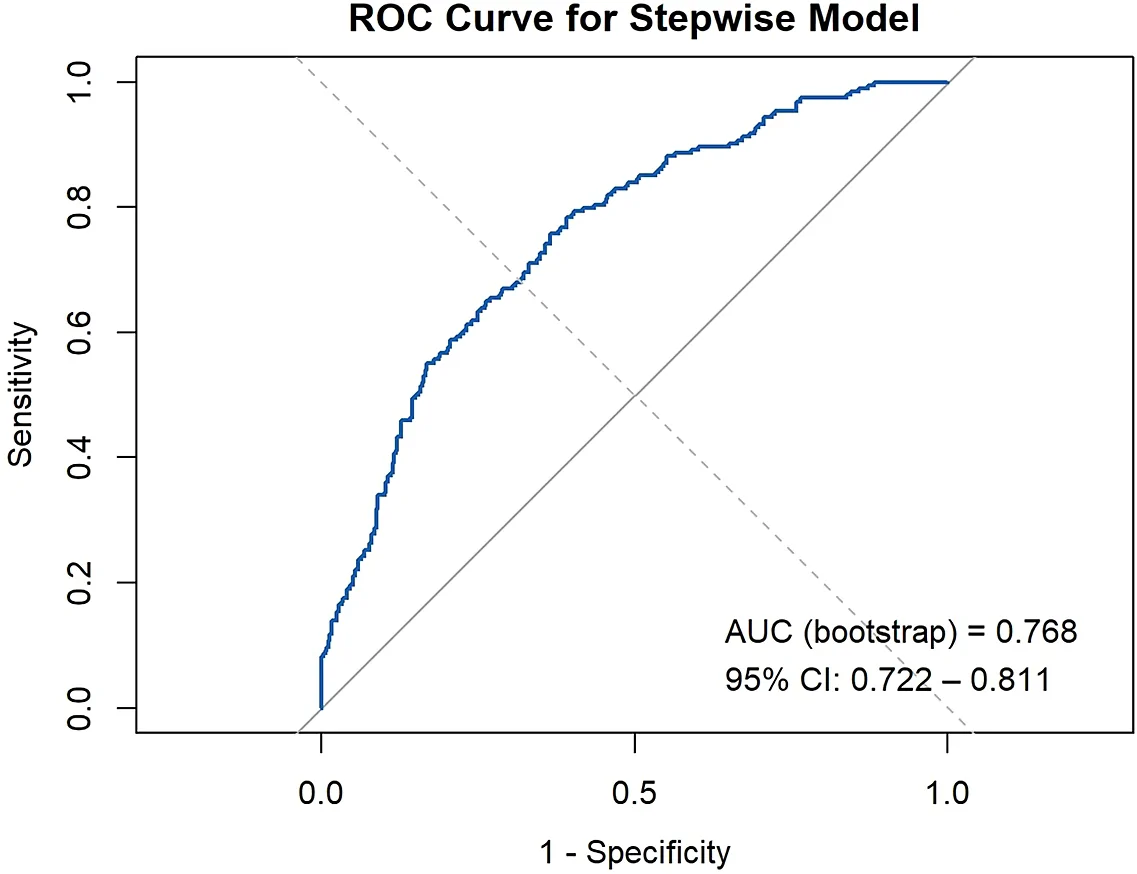
ROC for the final multivariate logistic regression model. The ROC curve shows the discrimination of the final model while the diagonal line indicates performance expected by chance. Model performance is summarized by the bootstrap-corrected AUC of 0.77 (95% CI 0.72–0.81). AUC, area under the receiver operating curve; CI, confidence interval; ROC, receiver operating curve.

On univariate analysis (**Supplementary Table 4**), higher age at presentation, higher OGTT glucose values, HbA1c at 24 to 28 weeks of GDM diagnosis, family history, prior GDM, Chinese ethnicity (vs Malay) and IVF use were associated with postpartum dysglycemia. In the final multivariate regression analysis (**Table 3**), family history of diabetes was associated with higher odds of postpartum dysglycemia (adjusted OR 2.31, 95%CI 1.56-3.42; p=0.00), as was prior GDM (adjusted OR 1.99, 95%CI 1.29-3.07; p=0.00),higher antenatal 2-hours OGTT glucose level (adjusted OR 1.22 per mmol/L, 95%CI 1.09-1.38; p=0.00) and higher antepartum HbA1c (adjusted OR 2.37 per % unit, 95%CI 1.51-3.79; p=0.00). IVF conception was associated with lower odds (adjusted OR 0.13, 95%CI 0.04-0.34; p=0.00) of postpartum dysglycemia, as was pre-pregnancy BMI (adjusted OR 0.93 per kg/m², 95%CI 0.89-0.96; p=0.00). This inverse association may reflect a relatively lean, β-cell predominant phenotype among women with postpartum dysglycemia. Compared with women of Malay ethnicity, women of Chinese ethnicity were associated with a higher odds (adjusted OR 1.92, 95%CI 1.16-3.22; p=0.01) of postpartum dysglycemia. Overall, the stepwise model demonstrated acceptable discrimination, with a bootstrap-corrected AUC of 0.77 (95%CI 0.72-0.81) (**Figure 1**).

**Table 3:** Final multivariate regression model generated.

Variable	OR (95% CI)	p-value
Age at presentation (years)	1.04 (0.99–1.09)	0.12
Race (Malay as reference)		
Chinese vs Malay	1.92 (1.16–3.22)	0.01
Indian vs Malay	1.32 (0.66–2.60)	0.43
Others vs Malay	0.94 (0.43–1.98)	0.88
2-hour glucose during OGTT at 24–28 weeks (mmol/L)	1.22 (1.09–1.38)	0.00
HbA1c at 24 to 28 weeks (%)	2.37 (1.51–3.79)	0.00
Pre-pregnancy BMI (kg/m^2^)	0.93 (0.89–0.96)	0.00
Family history of diabetes	2.31 (1.56–3.42)	0.00
PMH of GDM	1.99 (1.29–3.07)	0.00
IVF status	0.13 (0.04–0.34)	0.00

BMI, body mass index; CI, confidence interval; HbA1c, glycated *hemoglobin*; GDM, gestational diabetes mellitus; IVF, *in-vitro* fertilization; OR, odds ratio; OGTT, oral glucose tolerance test; PMH, past medical history.

Variables comprise of age at presentation, ethnicity (Malay as reference), 2-hours glucose during OGTT at 24–28 weeks, HbA1c at 24 to 28 weeks; pre-pregnancy BMI; family history of diabetes; previous history of GDM; and IVF status. Adjusted ORs, 95% CIs and p-values for each variable are presented. Statistically significance was set at p<0.05.

### 3.4 Dominance analysis to assess relative contribution of predictors

We used dominance analysis to assess the contribution of each predictor to our regression model. The 2-hour OGTT glucose level at 24 to 28 weeks was the most significant contributor in the multivariate model for postpartum dysglycemia (normalized mean contribution: 18.8%, 95%CI 15.0%-22.6%), followed by family history of diabetes (17.1%, 95%CI 13.6%-20.5%). Other variables ranked lower: antepartum HbA1c at 24 to 28 weeks (13.9%, 95%CI 11.1%-16.6%), pre-pregnancy BMI (13.3%, 95%CI 10.6%-15.9%), IVF status (12.6%, 95%CI 10.1%-15.0%), ethnicity (11.1%, 95%CI 8.9%-13.4%), and prior GDM (10.3%, 95%CI 8.2%-12.3%), with age demonstrating the smallest contribution (3.1%, 95%CI 2.4%-3.7%) (**Table 4**).

**Table 4:** Dominance analysis quantifying relative contribution of variables in the final model for predicting postpartum dysglycemia.

Variable	Normalized mean contribution (95% CI)
Age at presentation (years)	3.1% (2.4%–3.7%)
Race	11.1% (8.9%–13.4%)
Antepartum OGTT 2hr glucose threshold (mmol/L)	18.8% (15.0%–22.6%)
Family history of diabetes	17.1% (13.6%–20.5%)
HbA1c at 24 to 28 weeks (%)	13.9% (11.1%–16.6%)
Pre-pregnancy BMI (kg/m^2^)	13.3% (10.6%–15.9%)
IVF status	12.6% (10.1%–15.0%)
PMH of GDM	10.3% (8.2%–12.3%)

BMI, body mass index; CI, confidence interval; HbA1c, glycated *hemoglobin*; GDM, gestational diabetes mellitus; IVF, *in-vitro* fertilization; OGTT, oral glucose tolerance test; PMH, past medical history.

Dysglycemia refers to abnormal glucose tolerance on postpartum OGTT, including impaired fasting glucose, impaired glucose tolerance or diabetes mellitus. Values represent the normalized mean contribution of each predictor to overall model fit, summing to 100%, with 95% CIs derived from bootstrap resampling. Higher percentages indicate greater relative contribution to model performance.

### 3.5 Antepartum thresholds for prediction of persistent postpartum dysglycemia

A 2-hour OGTT threshold of 8.5 mmol/L at 24 to 28 weeks antepartum was an optimal threshold for stratifying risk of postpartum dysglycemia. Higher 2-hour OGTT glucose values are likely to capture a continuum of glycemic severity associated with a greater likelihood of persistent postpartum dysglycemia in women with GDM (**Supplementary Table 5**). However, this 2-hour glucose level is essentially identical to the diagnostic threshold for GDM, suggesting limited additional clinical value when used as a standalone triage tool. Consistent with this, the 2-hour OGTT threshold had low sensitivity (0.41, 95%CI 0.34-0.48) but higher specificity (0.81, 95%CI 0.77-0.85) for predicting postpartum dysglycemia. Conversely, an antepartum HbA1c threshold of 5.45% showed high sensitivity (0.77, 95%CI 0.71-0.83) but lower specificity (0.42, 95%CI 0.38-0.47), supporting its utility as a clinical threshold to identify women who progress to postpartum dysglycemia after delivery.

## 4 Discussion

In this multiethnic cohort managed within a structured postpartum pathway, early postpartum dysglycemia was common, with 34.2% of women with GDM having persistent dysglycemia (80% prediabetes, 20% DM) at a median of 6.8 weeks after delivery. Importantly, 28.9% of women missed the postpartum OGTT, despite integration of OGTT into routine postnatal obstetric follow-up, highlighting a persistent implementation gap even in settings with organized care transitions. Among women who underwent OGTT, persistent dysglycemia after delivery was more common in women of Chinese ethnicity and less frequent in women of Malay ethnicity compared to the NGT group. Notably, women who had postpartum dysglycemia were leaner, with lower pre- and mid-pregnancy BMI despite similar gestational weight gain with the NGT group. Despite being leaner, these women had higher antepartum HbA1c and 2-hour OGTT glucose levels. Postpartum dysglycemia appeared to be driven less by differences in insulin resistance and more by a relative reduced β-cell function, reflected by lower HOMA-β.

These findings reinforce the emerging view that GDM and the post-GDM state are metabolically heterogeneous, and a uniform approach to risk assessment may overlook clinically important subgroups. Powe et al. (33) defined physiological subtypes of GDM using the Matsuda index based on an antepartum OGTT and 51% of women with GDM had predominant insulin resistance, 30% insulin secretion defect, whereas 20% had both mixed insulin resistance and insulin secretion defects. In our cohort, the inverse association between pre-pregnancy BMI and postpartum dysglycemia, together with the 13.3% contribution of BMI to model performance, supports the presence of a relatively lean, β-cell–predominant phenotype. This inverse association should not be interpreted as indicating that higher BMI is protective; rather, it may reflect a subgroup in whom impaired β-cell reserve contributes more strongly to postpartum dysglycemia than generalized adiposity or insulin resistance. Stratification by pre-pregnancy BMI also showed a greater proportion of women of Chinese ethnicity in normal/underweight categories compared with women of Malay ethnicity (**Supplementary Table 3**). This further supports an Asian “lean high-risk” subgroup in whom β-cell dysfunction and visceral adiposity may be inadequately reflected by BMI alone (17, 18).

Our results align with studies in Asian populations demonstrating that reduced insulin secretion often predominates on a background of modest insulin resistance. A cross-sectional study in Korea showed that 20% of people with T2DM had moderate to severe insulin secretory defects, characterized by fasting serum C-peptide ≤1.7 ng/mL (34). Dynamic metabolic testing (OGTT, intravenous glucose challenge, and hyperinsulinaemic–euglycemic clamp) had shown marked impairments in acute insulin response and β-cell responsivity among Asians with dysglycemia who had mild insulin resistance (19). In post-GDM cohorts, β-cell impairment was strongly associated with postpartum hyperglycemia in lean women (OR 13.6, 95%CI 4.06-45.3), whereas insulin resistance was more relevant in women who had obesity (OR 45.8, 95%CI 18.5-113) (19). Reduced β-cell function appears to predispose Asians to T2DM (35), and preventive strategies may need to consider approaches that increase insulin secretion (36) or preserve β-cell function, such as short-term intensive insulin therapy for newly diagnosed T2DM (37, 38).

*In-vitro* fertilization (IVF) conception was inversely associated with postpartum dysglycemia. However, due to the small number of IVF pregnancies in our cohort, this observation should be interpreted cautiously. This apparent association should be considered hypothesis-generating and warrants confirmation in larger datasets. In our multivariate logistic regression and dominance analysis, postpartum dysglycemia was most strongly driven by the antepartum glycemic indices. The antepartum 2-hour OGTT glucose level and HbA1c suggest that women who manifest higher post-load glucose levels and those with chronic hyperglycemia during pregnancy are at the highest risk of persistent dysglycemia after delivery. In addition, family history of diabetes and prior GDM were also predictive of postpartum dysglycemia. This is concordant with previous studies (39, 40) and likely reflects underlying inherited risk and β-cell vulnerability. Using Youden’s index, an antepartum HbA1c threshold of 5.45% yielded a sensitivity of 77% and specificity of 42% for postpartum dysglycemia. Likewise, Varejão et al. reported that women with third-trimester HbA1c≥5.4% had higher odds of developing postpartum diabetes (41). Kwon et al. (42) reported a HbA1c cut-off of 5.55% with 78.6% sensitivity and 72.5% specificity for predicting postpartum diabetes at 3 months of follow-up. In Sweden, Claesson et al. (43) showed that a lower HbA1c cut-off of 5.4% was associated with 5-fold increased odds of diabetes at five years after pregnancy. The high positive predictive value (0.81, 95%CI 0.77-0.85) and the low negative predictive value (0.4, 95%CI 0.33-0.47) of our HbA1c threshold support its clinical use as a risk stratification tool rather than a standalone rule-out test. Furthermore, modest differences in mean HbA1c may reflect racial variation in erythrocyte turnover (44, 45), *hemoglobin* glycation, red cell survival (46) and or altered iron metabolism (47). Discordance between HbA1c and glucose tolerance test results are also more prevalent in non-Caucasian ethnic groups (48). These considerations are particularly relevant in ethnically diverse populations, although women with *hemoglobin* variants known to interfere with HbA1c measurement were excluded from the present study. Ethnic differences in dysglycemia may also be influenced by differential screening uptake rates. Prior studies in 11825 women with GDM-complicated pregnancies suggest the odds of postpartum glucose testing were higher for Asian women compared to women of Hispanic or White ethnicity (49) and we have previously reported a higher-odds of postpartum OGTT uptake among women of Chinese ethnicity (16). These findings emphasize the need for tailored postpartum strategies that combine risk prediction variables with targeted intervention to prevent diabetes.

This study demonstrates standard care practices in a tertiary center following an organized postpartum protocol. It also includes thorough antepartum and postpartum phenotyping along with a strong modelling approach that uses multiple imputation, bootstrap analysis for internal variability, and dominance analysis to measure the impact of predictors. However, this study has several limitations. First, its retrospective single-center design may limit generalizability to other settings. Second, postpartum glycemic status could only be determined in women who completed the postpartum OGTT. Although 28.9% did not complete testing, this reflects follow-up within a real-world clinical service rather than a prospective study with mandated research visits. Additional study-directed tracing or testing was therefore not undertaken. As some antepartum characteristics differed between completers and non-completers, the prevalence and prediction estimates should be interpreted as applying primarily to women with an observed postpartum glycemic outcome (**Supplementary Table 2**). The retrospective design of the study also limited measurement of several clinically relevant postpartum factors such as breastfeeding intensity, postpartum depression, and dietary intake, which were not consistently documented in clinical records. Chronic low-grade inflammation contributes to insulin resistance, β-cell dysfunction and diabetes progression (50); however, inflammatory biomarkers were not systematically measured in this present cohort. Socioeconomic status (SES) was assessed in our preliminary analyses, but it did not affect outcomes. As Singapore’s public housing and land-use policies are designed to promote socioeconomic mixing across residential districts (51) and to preserve degrees of freedom and avoid overfitting, SES was excluded from the final analysis.

## 5 Conclusion

Early postpartum dysglycemia was frequent following GDM in this multiethnic Asian cohort and was characterized by a lean phenotype with relative β-cell dysfunction. A pragmatic prediction model using routinely collected antepartum variables showed acceptable discrimination and identified clinically actionable thresholds that may support tiered postpartum surveillance. External validation in independent, multi-ethnic Asian cohorts are needed before routine clinical implementation to determine whether risk-based triage can improve postpartum OGTT uptake and facilitate earlier, targeted diabetes prevention.

## Data Availability

The data analyzed in this study is subject to the following licenses/restrictions: The datasets generated and/or analyzed during the current study are not publicly available due to ethical restrictions but are available from the corresponding author on reasonable request. Requests to access these datasets should be directed to p.eng@nus.edu.sg.
