# Supplementary material for: Early postpartum dysglycemia after gestational diabetes: β-cell dysfunction and risk stratification in a multiethnic cohort
Source: Front Endocrinol (Lausanne). 2026 Aug 12;17:1913417. doi: 10.3389/fendo.2026.1913417 (PMC13506360; doi:10.3389/fendo.2026.1913417)
Supplement: Supplementary file 1 [file Table1.docx]

**Supplementary Table 1.** Baseline prepartum and antepartum characteristics of women in our study stratified by IVF use. Continuous variables are presented as median (IQR). Categorical variables are presented as frequency (%). Group comparisons were made using the Mann–Whitney U test for continuous variables and the χ² or Fisher’s exact test for categorical variables, as appropriate. Statistical significance was set at p < 0.05

| **Variable** | **IVF Status** | | **p-value** |
| --- | --- | --- | --- |
|  | **No (n = 518)** | **Yes (n = 36)** |  |
| Age at presentation (years) | 33 (30, 36) | 37 (35, 39.3) | 0.00* |
| Gravidity | 2 (1, 3) | 2 (1, 3) | 0.4 |
| Parity | 1 (1, 2) | 1 (1, 1) | 0.5 |
| Pre-pregnancy weight (kg) | 65 (56, 76.6) | 60 (50, 68.5) | 0.03* |
| Pre-pregnancy BMI (kg/m2) | 26 (22.5, 30.8) | 22.9 (20.4, 27.1) | 0.01* |
| Weight at 24-28 weeks (kg) | 70 (60.4, 81.7) | 70 (62.6, 75.9) | 0.77 |
| BMI at 24-28 weeks (kg/m2) | 26 (22.5, 30.8) | 27.8 (20.4, 27.1) | 0.74 |
| Weight at delivery (kg) | 74 (64.7, 87) | 69.2 (62.5, 80.3) | 0.23 |
| BMI at delivery (kg/m2) | 30 (25.9, 34.3) | 27.8 (25.5, 32.9) | 0.11 |
| HbA1c at GDM diagnosis (%) | 5.2 (4.9, 5.5) | 5.2 (5, 5.6) | 0.97 |
| Baseline glucose during OGTT at 24-28 weeks (mmol/L) | 4.6 (4.3, 5.1) | 4.6 (4.2, 5.2) | 0.97 |
| 1hr glucose during OGTT at 24-28 weeks (mmol/L) | 10.2 (9.3, 11) | 10 (9.4, 10.8) | 0.79 |
| 2hr glucose during OGTT at 24-28 weeks (mmol/L) | 8.8 (7.9, 9.6) | 9.4 (8.4, 10.3) | 0.97 |
| FH of DM | 196 (45.3) | 8 (22.2) | 1 |
| PMH of GDM | 130 (25.7) | 5 (13.9) | 0.17 |
| PMH of MASLD | 1 (0.2) | 1 (0.4) | 1 |
| IVF use | 4 (2.9) | 0 (0) | 1 |
| Ethnicity | | | |
| Chinese | 214 (41.4) | 18 (50) | 0.31 |
| Malay | 194 (37.5) | 18 (50) |  |
| Indian | 59 (11.4) | 6 (16.7) |  |
| Others | 50 (9.7) | 16 (45.7) |  |
| GDM treatment | | | |
| Diet | 358 (69.9) | 29 (80.6) | 0.54 |
| Insulin | 137 (26.8) | 6 (16.7) |  |
| Metformin | 13 (2.5) | 1 (2.8) |  |
| Insulin + Metformin | 4 (0.8) | 0 (0) |  |

*Abbreviations: IQR, interquartile range; IVF, in-vitro fertilisation; BMI, body mass index; OGTT, oral glucose tolerance test; FH, family history; PMH, past medical history; DM, diabetes mellitus; GDM, gestational diabetes mellitus; MASLD, metabolic dysfunction-associated steatotic liver disease*

**Supplementary Table 2** Comparison of antepartum characteristics between women who completed and did not complete the postpartum oral glucose tolerance test. Continuous variables are presented as median (25th percentile, 75th percentile), and categorical variables as n/N (%), where N represents the number of participants with available data. Between-group comparisons were performed using the Mann–Whitney U test for continuous variables and the χ² test or Fisher’s exact test for categorical variables, as appropriate. Statistical significance was set at p < 0.05.

| **Variable** | **Attendance at postpartum OGTT testing (n = 409)**  **Median (IQR) or n/n (%)** | **Non-attendance of postpartum OGTT testing (n = 166)**  **Median (IQR) or n/n (%)** | **P-value** |
| --- | --- | --- | --- |
| Age at presentation (years) | 33 (30, 37) | 33 (30, 37) | 0.67 |
| Ethnicity (%)  Chinese  Malay Indian  Others | 204/408 (50)  130/408(31.9) 40/408 (9.8)  34/408 (8.3) | 34/159 (21.4)  76/159(47.8) 28/159 (17.6)  21/159 (13.2) | 0.00 |
| 2-hour glucose during OGTT at 24-28 weeks (mmol/L) | 8.8 (7.9, 9.5) | 9 (7.7, 10) | 0.42 |
| HbA1c at 24 to 28 weeks (%) | 5.2 (4.9, 5.5) | 5.3 (5, 5.7) | 0.00 |
| Pre-pregnancy BMI (kg/m2) | 25.2 (21.6, 29.4) | 28.6 (25, 33) | 0.00 |
| Family history of diabetes | 178/367 (48.5) | 37/109 (33.9) | 0.01 |
| PMH of GDM | 95/402 (23.6) | 42/144 (29.2) | 0.23 |
| IVF status | 29/406 (7.1) | 7/148 (4.7) | 0.41 |

*Abbreviations: BMI, body mass index; GDM, gestational diabetes mellitus; HbA1c, glycated haemoglobin; IVF, in vitro fertilisation; OGTT, oral glucose tolerance test; PMH, past medical history.*

**Supplementary Table 3.** Racial distribution of women who developed postpartum dysglycaemia (n = 140) stratified by pre-pregnancy BMI.

| **Ethnicity** | **Pre-pregnancy BMI (kg/m^2^) †** | | | | **p-value** |
| --- | --- | --- | --- | --- | --- |
|  | **Underweight (n = 11)** | **Normal**  **(n = 45)** | **Overweight**  **(n = 32)** | **Obese**  **(n = 44)** |  |
| Malay | 2 (18.2) | 3 (6.7) | 8 (25) | 21 (47.7) | 0.00* |
| Chinese | 8 (72.7) | 37 (82.2) | 19 (59.4) | 18 (40.9) |  |
| Indian | 1 (9.1) | 1 (2.2) | 3 (9.4) | 4 (9.1) |  |
| Others | 0 (0) | 4 (8.9) | 2 (6.3) | 1 (2.3) |  |

*Abbreviations: BMI, body mass index*

† *Patient stratification was based on Asian-specific BMI categories: underweight (<18.5kg/m2), normal (18.5 – 22.9kg/m2), overweight (23 – 27.4kg/m2), obese (****≥****27.5kg/m2)*

**Supplementary Table 4.** Univariate logistic regression analyses for all imputed variables following multiple imputation. Unadjusted odds ratio, 95% CI and p-value for each variable are presented. Statistical significance was set at p<0.05.

| **Variable** | **OR (95% CI)** | **p-value** |
| --- | --- | --- |
| Age at presentation (years) | 1.04 (1.01–1.09) | 0.03* |
| Gravidity | 1.00 (0.89–1.13) | 0.94 |
| Parity | 1.05 (0.90–1.22) | 0.51 |
| Prepartum weight (kg) | 0.98 (0.97–0.99) | 0.00* |
| Prepartum BMI (kg/m2) | 0.95 (0.92–0.97) | 0.00* |
| Antepartum weight (kg) | 0.98 (0.97–0.99) | 0.00* |
| Antepartum BMI (kg/m2) | 0.95 (0.92–0.98) | 0.00* |
| Delivery weight (kg) | 0.98 (0.97–0.99) | 0.00* |
| Delivery BMI (kg/m2) | 0.96 (0.93–0.98) | 0.00* |
| Change in weight over pregnancy (kg) | 1.01 (0.99–1.04) | 0.23 |
| Change in BMI over pregnancy (kg/m2) | 1.03 (0.97–1.10) | 0.31 |
| Baseline glucose during OGTT at 24-28 weeks (mmol/L) | 1.29 (1.06–1.58) | 0.01* |
| 1hr glucose during OGTT at 24-28 weeks (mmol/L) | 1.17 (1.06–1.3) | 0.00** |
| 2hr glucose during OGTT at 24-28 weeks (mmol/L) | 1.32 (1.19–1.47) | 0.00* |
| HbA1c at 24 to 28 weeks of GDM diagnosis (%) | 1.99 (1.41–2.85) | 0.00* |
| FH of DM | 2.32 (1.63–3.31) | 0.00* |
| PMH of GDM | 2.14 (1.45–3.16) | 0.00* |
| PMH of PCOS | 1.54 (0.72–3.22) | 0.26 |
| PMH of MASLD | 0.00 (NA–NA) | 0.98 |
| IVF use | 0.27 (0.09–0.64) | 0.01* |
| Ethnicity (Malay as reference) | | |
| Chinese | 2.13 (1.43–3.19) | 0.00* |
| Indian | 1.22 (0.66–2.20) | 0.51 |
| Others | 1.14 (0.58–2.17) | 0.7 |

*Abbreviations: OR, odds ratio; CI, confidence interval; BMI, body mass index; OGTT, oral glucose tolerance test; HbA1c, glycated haemoglobin; FH, family history; PMH, past medical history; DM, diabetes mellitus; GDM; gestational diabetes mellitus; PCOS, polycystic ovary syndrome; MASLD, metabolic dysfunction-associated steatotic liver disease; IVF, in-vitro fertilisation*

**Supplementary Table 5.** ROC-derived optimal cutoff points for key continuous predictors identified using the Youden index. Sensitivity, specificity, PPV and NPV are presented with 95% CIs

| **Variable** | **Threshold** | **Sensitivity (95% CI)** | **Specificity (95% CI)** | **PPV (95% CI)** | **NPV (95% CI)** |
| --- | --- | --- | --- | --- | --- |
| HbA1c at GDM diagnosis (%) | 5.45 | 0.77 (0.71–0.83) | 0.42 (0.38–0.47) | 0.73 (0.66–0.8) | 0.48 (0.43 - 0.53) |
| 2hr glucose during OGTT at 24-28 weeks (mmol/L) | 8.45 | 0.41 (0.34–0.48) | 0.81 (0.77–0.85) | 0.81 (0.77–0.85) | 0.40 (0.33–0.47) |

*Abbreviations: ROC, receiver operating curve; CI, confidence interval; GDM, gestational diabetes mellitus; HbA1c, glycated haemoglobin; OGTT, oral glucose tolerance test; PPV, positive predictive value; NPV, negative predictive value*
